# Melatonin protects against blood-brain barrier damage by inhibiting the TLR4/ NF-κB signaling pathway after LPS treatment in neonatal rats

**DOI:** 10.18632/oncotarget.15780

**Published:** 2017-02-28

**Authors:** Yingying Hu, Zhouguang Wang, Shulin Pan, Hongyu Zhang, Mingchu Fang, Huai Jiang, Hao Zhang, Zhengzheng Gao, Kebin Xu, Zhenmao Li, Jian Xiao, Zhenlang Lin

**Affiliations:** ^1^ Department of Neonatology, The Second Affiliated Hospital & Yuying Children's Hospital, Wenzhou Medical University, Wenzhou, Zhejiang 325000, China; ^2^ Molecular Pharmacology Research Center, School of Pharmacy, Wenzhou Medical University, Wenzhou, Zhejiang 325035, China

**Keywords:** blood-brain barrier, melatonin, TLR4/NF-κB, white matter injury, hypoxic-ischemic and inflammatory

## Abstract

Hypoxic-ischemic and inflammatory (HII) induces the disruption of blood–brain barrier (BBB) which leads to inflammatory responses and neuronal cell death, resulting in brain secondary damage. Previous studies showed that melatonin produced potent neuroprotective effects in neonatal hypoxic-ischaemic models. However, the relationship between BBB disruption and melatonin in HII was still unclear. The present study therefore investigated the beneficial effects of melatonin on BBB after HII and the underlying mechanisms. HII animal model was conducted by receiving lipopolysaccharide followed by 90 min hypoxia-ischaemia in postnatal day 2 Sprague–Dawley rat pups. Melatonin was injected intraperitoneally 1 h before lipopolysaccharide injection and then once a day for 1 week to evaluate the long-term effects. In this study, we demonstrated that melatonin administration inhibited the disruption of BBB permeability and improved the white matter recovery in HII model rats. Melatonin significantly attenuated the degradation of junction proteins and the neuroprotective role was related to the inhibition of microglial toll-like receptor 4/ nuclear factor-kappa B signaling pathway both *in vivo* and *in vitro*. Taken together, our data demonstrated that therapeutic strategies targeting inflammation might be suitable for the therapy of preserving BBB integrity after HII.

## INTRODUCTION

Cerebral white matter injury (WMI) is a common and leading cause of brain injury that often results in chronic neurologic disabilities including cerebral palsy, cognitive/behavioural/attentional deficits in premature infants [1, 2]. These chronic neurologic disabilities create devastating health consequences imposing a substantial economic burden worldwide. However, despite much time and extensive effort, there is lack of successful intervention for treating WMI associated neurological diseases. Currently, hypothermia is a therapeutic strategy, but comes along with undesirable side effects, consisting of uncompleted neuroprotection and abnormal neurodevelopment [3]. Thus, new therapies are desperately needed.

Several lines of preclinical and clinical evidence suggest that hypoxic ischemic-induced inflammation (HII) during early brain development is the cause leading to white matter damage [4]. During the perinatal period, the brain's white matter is thought to be the most susceptible region to inflammatory injury at the time of, or just prior to, the beginning of myelination process. It is widely accepted that a profound brain injury induced by neuroinflammation is, at least partially, a result of increased permeability of the blood–brain barrier (BBB) in response to systemic inflammation [5, 6]. The BBB is formed by specialized endothelial cells lining the cerebral microvasculature, with a protective function in the control of molecular traffic between blood and the brain. Thus, the BBB plays an important role in the homeostatic regulation of the brain's internal environment [7]. It should be noted that the accessory structure is an integral component of the BBB, which consists of the basement membrane, pericytes, and astrocytic endfeet. The structural integrity of the BBB is crucial to ensure proper neuronal function and protects the central nervous system (CNS) from injury and disease. [8]. WMI can be caused by inflammatory insult from early pregnancy, when a robust production of pro-inflammatory cytokines disrupts vasculature integrity of the BBB. Initial leukocyte recruitment at the injury site was followed by monocyte/macrophage infiltration. Overall, a sustained neuroinflammation is a common feature of intracerebral hemorrhage, leading to onset of WMI [9].

Toll-like receptors (TLRs) are a class of cell surface protein that plays a key role in the innate immune responses. Among all TLRs, the TLR4 is recognized as a sensing receptor to lipopolysaccharide (LPS), a cell wall component from Gram-negative bacteria [9–10]. Mechanically, The LPS-induced inflammation is mediated by activation of the TLR4 and its downstream MAPK signaling cascade. The TLR4-MAPK pathway promotes nuclear translocation of the nuclear factor-kappa B (NF-κB) transcription factor. Binding of NF-κB to DNA markedly enhances production of pro-inflammatory factors [11]. TLR4 is widely expressed by microglia, and thus it is not surprising that the TLR4 downstream signaling cascade is also involved in neuroinflammatory diseases [9]. A recent study using, a mouse model of intrauterine infection showed that the TLR signaling induced preterm labor, which was mediated by activation of the NF-κB pathway [12, Furthermore, mice with MyD88 knockout showed improved LPS-sensitized hypoxic ischemic-induced brain injury, and this protective effect was likely mediated by reduced NF-κB activation and attenuated pro-inflammatory cytokine and chemokine production [13].

Melatonin (N-acetyl-5-methoxytryptamine) is mainly produced from the pineal gland. Previous studies have found that melatonin has a diverse pharmacological activity, ranges from anti-oxidation, anti-inflammation to anti-apoptotic effects [14–16]. he BBB is permeable to melatonin with no potential harms [17]. Accumulating evidence supports that melatonin has a protective effect against experimental cerebral ischemic and reperfusion damage in rodents. These studies showed that treatment of melatonin reduced infarct size and improved functional outcomes in adult mice with middle cerebral artery occlusion (MCAO) and stroke. A similar therapeutic effect was observed in neonatal HI models [18–20]. Recently, several studies demonstrated that treatment of melatonin improved WMI in neonatal animals [7], However, the molecular mechanism by which melatonin regulates the BBB function in the setting of WMI is unknown. In this study, we designed to investigate the effects of melatonin and the involvement of neuroinflammation on BBB disruption after neonatal WMI both *in vivo* and *in vitro*.

## RESULTS

### Treatment of melatonin improves white matter recovery and reduces BBB permeability in a neonatal model of HII

**Figure 1:**
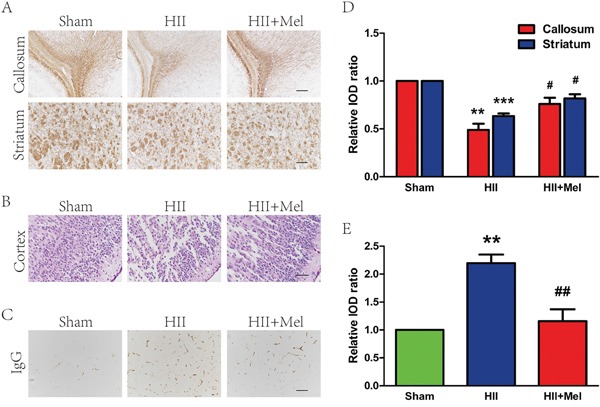
Melatonin improved white matter recovery and reduced BBB permeability after neonatal HII **A**. MBP staining in the white matter of callosum and striatum regions at 21 d after HII. Scale = 400 μm (callosum) and 200 μm (striatum). **B**. HE staining in the brain of cortex region at 24 h after HII. Scale bar = 100 μm. **C**. BBB permeability was evaluated by serum IgG extravasation in rats 24 h after HII. Scale bar = 200 μm. **D**. Analysis of the immunohistochemistry results from A. **P < 0.01, ***P < 0.001 versus the Sham group. ^#^*P* < 0.05 versus the HII group. Mean values ± SEM, n = 6 rats per group. **E**. Quantification of BBB permeability data from C by software Image-pro Plus. **P < 0.01 versus the Sham group. ^##^*P* < 0.01 versus HII group. Mean values ± SEM, n = 5 rats per group.

To evaluate the therapeutic effects of melatonin on HII, rats were intraperitoneally administered with melatonin 1 h before injury and further treated on a daily basis for 1 week. In rodents, remyelination begins at 7 to 14 days post-lesion, depending on the location of the lesion and the age of the animal and most of the remyelination is essentially complete by 3–4 weeks [21]. Neonatal rats with HII displayed hypomyelination when compared to sham animals. A closed examination on myelin sheaths showed a significant impairment in brain development with disrupted structure in the HII group. The HII group received melatonin treatment had significantly higher expression of MBP than the untreated group. Interestingly, myelin structure appears to be normal in melatonin-treated group (Figure 1A). Morphology of the cortex was further examined by HE staining. Treatment of melatonin significantly increased the myelination in the HII group, whereas saline-treated HII-induced neonatal rats displayed grey matter damage, as characterized by shrunken neurons containing pale homogenous cytoplasm (Figure 1B). The sham rats showed integrated infrastructures, clear boundary, morphologically normal neurons with a clear cytoplasm, and uniform and clear nuclei in the cortex. Immunoglobulin G (IgG) extravasation was used as an indicator of BBB permeability [22, 23]. Importantly, melatonin treatment showed a neuroprotective effect in the cortex as a significant reduction of IgG expression was observed in HII-induced mice (Figure 1C). The effect of melatonin on BBB permeability was evaluated by IgG staining at 24 h after injury. As shown in Figure 1D and 1E, BBB permeability was statistically significantly increased in response to HII as compared with uninjured sham control, but reduced by treatment with melatonin. Taken together, these results suggest that treatment of melatonin could effectively improves white matter recovery and prevent BBB disruption in HII-induced neonatal rats.

### Treatment of melatonin prevents the loss of tight junction and adherens unction proteins after neonatal HII

**Figure 2:**
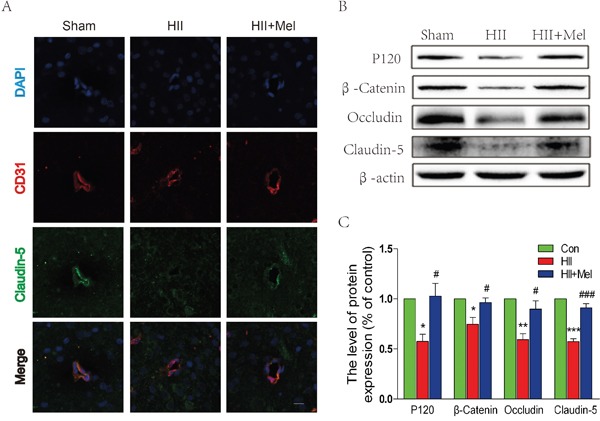
Melatonin prevented the loss of tight junction and adherens junction proteins after neonatal HII **A**. Representative micrographs showing double immunofluorescence with Claudin-5 (green) and CD31 (endothelial cell marker, red), nuclei were labeled with DAPI (blue) in each group. Scale bar = 75 μm. **B**. Representative western blots of adherens junction proteins (β-Catenin and P120) and tight junction proteins (Occludin and Claudin-5) in the sham, HII model and HII model treated melatonin groups 24 h after HII. **C**. Quantification of western blot data from B. *P < 0.05, **P < 0.01, ***P < 0.001 versus the Sham group. ^#^*P* < 0.05, ^###^*P* < 0.001 versus HII group. Mean values ± SEM, n = 5 rats per group.

The tight junction (TJ) and adherens junction (AJ) seal adjacent endothelial cells (ECs) of blood vessels, and thus they play an important role in the regulation of BBB integrity and permeability [24–26]. We sought to determine whether melatonin prevents the loss of these junctions after neonatal HII. To directly investigate the endothelial tight junction, we performed double immunostaining for CD31, an EC-specific marker, and claudin-5, a TJ-specific marker in the ipsilateral brain of neonatal rats. Our results demonstrated reduced staining for claudin-5 expression in CD31^+^ cells in the saline-treated HII group, but no observable change in melatonin-treated HII group when compared the sham group (Figure 2A). These results were corroborated with the western blot analyses, in which expression levels of AJ proteins (P120-catenin and β-Catenin) and TJ proteins (occluding and claudin-5) were significantly decreased in the saline-treated HII group. Treatment of melatonin increased the AJ and TJ proteins to levels similar to those of the sham group (Figure 2B and 2C). These results demonstrate that melatonin preserves the structure integrity of BBB after HII, which at least is partly mediated by restoration of TJ and AJ protein expressions.

### Treatment of melatonin prevents the loss of pericytes after neonatal HII

**Figure 3:**
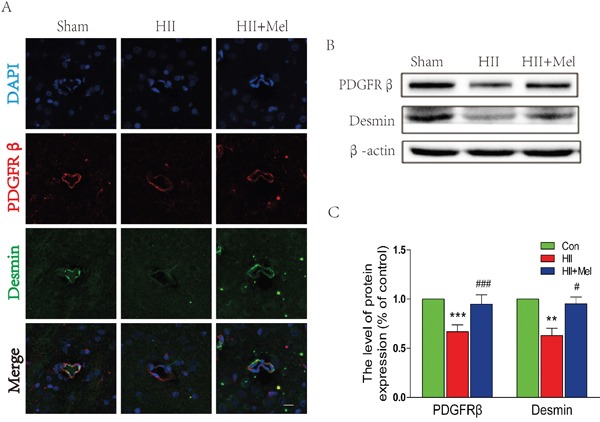
Melatonin prevented the loss of pericytes after neonatal HII **A**. Representative micrographs showing double immunofluorescence with Desmin (green) and PDGFRβ (red), nuclei were labeled with DAPI (blue) in each group. Scale bar = 75 μm. **B**. Representative western blots of pericyte markers PDGFRβ and Desmin in the sham, HII model and HII model treated melatonin groups 24 h after HII. **C**. Quantification of western blot data from B. **P < 0.01, ***P < 0.001 versus the Sham group. ^#^*P* < 0.05, ^###^*P* < 0.001 versus HII group. Mean values ± SEM, n = 5 rats per group.

Pericytes are located at intervals along the walls of capillaries (and post-capillary venules). In the CNS, they are important for blood vessel formation, maintenance of the BBB, as well as play an important role in the regulation of immune cell across the BBB from the peripheral [27]. PDGFRβ and desmin are two pericyte markers were used to detect the abundance of pericyte in brain. Immunostaining for PDGFRβ and desmin showed reduced numbers of pericytes in HII-induced neonatal rats (Figure 3A). Interestingly, treatment of melatonin significantly increased both PDGFRβ and desmin staining in the brain, and these observations were confirmed by the western blot, where protein levels of PDGFRβ and Desmin were markedly abolished in the HII group, but were restored by the treatment of melatonin in neonatal rats with HII (Figure 3B and 3C). Taken together, our results indicate that melatonin prevents BBB disruption is associated with increased pericyte survival after neonatal HII.

### Treatment of melatonin decreases astrogliosis and microgliosis after neonatal HII

**Figure 4:**
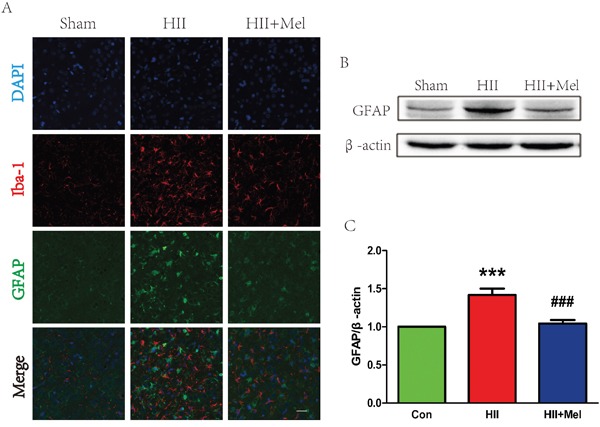
Melatonin decreased astrogliosis and microgliosis after neonatal HII **A**. Representative micrographs showing double immunofluorescence with GFAP (red) and Iba-1 (microglia marker, green), nuclei were labeled with DAPI (blue) in each group. Scale bar = 75 μm. **B**. Representative western blots of astrocytes marker GFAP in the sham, HII model and HII model treated melatonin groups 24 h after HII. **C**. Quantification of western blot data from B. ***P < 0.001 versus the Sham group. ^###^*P* < 0.001 versus HII group. Mean values ± SEM, n = 5 rats per group.

The LPS challenge is able to induce the activation of gliocytes in the brain. Our next question was to examine whether melatonin could abrogate LPS-induced activation of gliocytes. Higher levels of GFAP (the astrocyte marker) and Iba-1 (the microglia marker) were detected in the HII group, while both GFAP and Iba-1 were decreased significantly indicating the activation of gliocyte was substantially reduced by treatment of melatonin after HII in neonatal rats (Figure 4A). In contrast, these markers were barely detectable in HII-induced neonatal rats treated with melatonin. Increased GFAP and Iba-1 immunostainings were further corroborated by western blot analyses showing that protein levels of these markers were elevated in the HII group, with no difference in these markers between the melatonin-treated HII group and the sham group (Figure 4B and 4C). These data suggest that treatment of melatonin attenuated the activation of astrocytes and microglia in the setting of HII in neonatal rats.

### Treatment of melatonin inhibits the inflammatory process and suppresses the TLR4/NF-κB signaling pathway after neonatal HII

**Figure 5:**
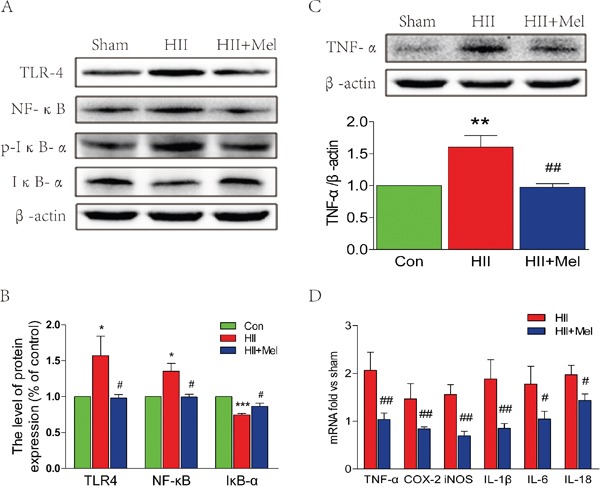
Melatonin suppressed TLR4/NF-κB signaling pathway proteins expression after neonatal HII **A**. Representative western blots of TLR4, NF-κB, p-IKB-α and IKB-α in the sham, HII model and HII model treated melatonin groups 24 h after HII. **B**. Quantification of western blot data from A. *P < 0.05, ***P < 0.001 versus the Sham group. ^#^*P* < 0.05 versus HII group. Mean values ± SEM, n = 5 rats per group. **C**. Representative western blot and quantification data of TNF-α in the sham, HII model and HII model treated melatonin groups 24 h after HII. β-actin was used as loading control. **P < 0.01 versus the Sham group. ^##^*P* < 0.01 versus HII group. Mean values ± SEM, n = 5 rats per group. **D**. RT-PCR analysis of RNA extracts from lesion side brains. Data were expressed as fold change versus sham-operated control. ^#^*P* < 0.05, ^##^*P* < 0.001 versus HII group. Mean values ± SEM, n = 6 rats per group.

It has been noted that inflammation is involved inneonatal HII [28]. Thus, the effect of melatonin on HII-induced inflammation was examined. Recent studies have demonstrated that TLR4 was an important mediator of neuroinflammation and tissue damage during infectious and non-infectious CNS diseases [29–31]. Western blot analyses showed that protein level of TLR4 was significantly increased, which was associated with enhanced levels of phosphorylated-IκB-α and NF-κB in the HII group treated saline (Figure 5A and 5B). However, the TLR4-NF-κB signaling pathway was significantly down-regulated in the HII group treated with melatonin, and was not changed as compared to the sham group (Figure 5A and 5B). Increased TNF-α protein expression was found in the HII group treated with saline, showing increased inflammatory response was induced by the LPS challenge in neonatal rats (Figure 5C). However, no significant changes in TNF-α were observed between the HII group treated with melatonin and the sham group, indicating that the inflammatory response was markedly suppressed by administration of melatonin (Figure 5C). The HII-induced neonatal rats treated with saline exhibited a one-fold increase in inflammatory factors, such as COX-2 and iNOS, and cytokines including TNF-α, IL-1β, IL-6 and IL-18 as compared to the melatonin-treated HII group (Figure 5D), suggesting treatment of melatonin treatment attenuates the inflammatory responses via inhibition of the TLR4/NF-κB signaling pathway after HII in neonatal rats.

### The decreased expression of TJ and AJ proteins induced by the LPS challenge is attenuated by treatment of melatonin *in vitro*

**Figure 6:**
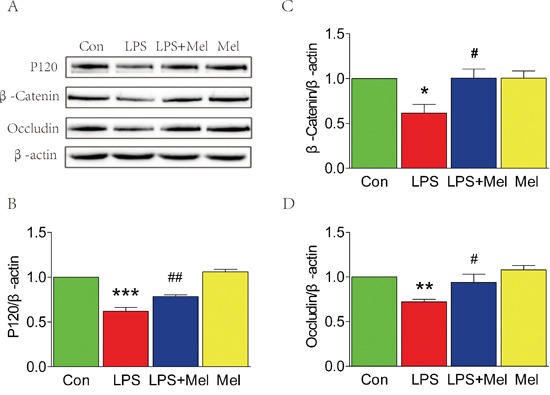
Melatonin attenuated the decreases of tight junction and adherens junction proteins induced by LPS in the transwell co-culture of BV-2 cells and HUVECs **A**. Representative western blots of tight junction and adherens junction proteins (β-Catenin, P120 and Occludin) in each group of HUVECs. **B., C**. and **D**. Quantification of western blot data from A. *P < 0.05, **P < 0.01, ***P < 0.001 versus the Control group. ^#^*P* < 0.05, ^##^*P* < 0.01 versus LPS group. Mean values ± SEM, n = 5 per group.

We used a transwell co-culture system, including both BV-2 cells and HUVECs, to examine the effects of microglia on ECs *in vitro*. Under the LPS challenge, protein level of P120, β-Catenin, and occludin were significantly down-regulated in HUVECs, but these changes were reversed by treatment of melatonin (Figure 6). It should be noted that melatonin alone did not lead to any changes in expression of TJ and AJ proteins when compared to the control group (Figure 6). Taken together, These results suggest that treatment of melatonin exerts a protective effect against the LPS challenge-mediated TJ and AJ dysfunctions in HUVECs.

### Treatment of melatonin suppresses activation of the TLR4/NF-κB signaling pathway in microglia *in vitro*

**Figure 7:**
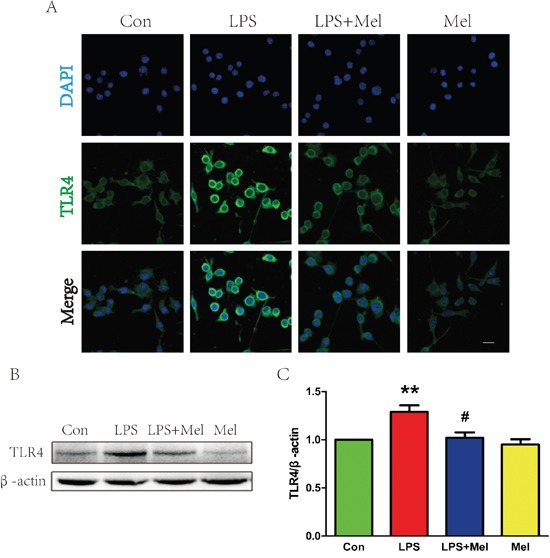
Melatonin decreased the expression of TLR4 protein induced by LPS in BV-2 cells **A**. Immunofluorescence staining of TLR4 (green) in BV-2 cells treated with LPS for 24 h, nuclei were labeled with DAPI (blue). Scale bar = 75 μm. **B**. Representative western blot of protein TLR4 in each group of BV-2 cells. **C**. Quantification of western blot data from B. **P < 0.01 versus the Control group. ^#^*P* < 0.05 versus LPS group. Mean values ± SEM, n = 5 per group.

**Figure 8:**
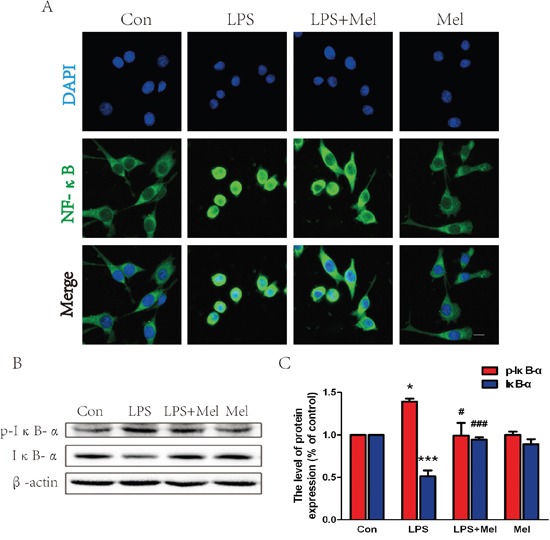
Melatonin inhibited NF-κB translocation induced by LPS in BV-2 cells **A**. Immunofluorescence staining for NF-κB translocation of BV-2 cells treated with LPS for 24 h, nuclei were labeled with DAPI (blue). Scale bar = 75 μm. **B**. Representative western blots of proteins p-IκB-α and IκB-α in each group of BV-2 cells. **C**. Quantification of western blot data from B. *P < 0.05, ***P < 0.001 versus the Control group. ^#^*P* < 0.05, ^###^*P* < 0.001 versus LPS group. Mean values ± SEM, n = 5 per group.

We investigated the effects of melatonin on the TLR4 downstream signaling pathways *in vitro*. When BV-2 cells stimulated by LPS, enhanced immunofluorescence staining for TLR4 was observed (Figure 7A). However, co-treatment of melatonin significantly reduced cell surface expression of TLR4 in BV-2 cells (Figure 7A). These findings were consistent with the western blot analyses showing that treatment melatonin inhibits elevated protein expression of TLR4 induced by the LPS challenge (Figure 7B and 7C). The downstream signaling pathway of TLR4 was further examined by double immunofluorescence staining and western blot (Figure 8). It is found that NF-κB nucleus translocation was significantly increased by the LPS challenge, with enhanced phosphorylation of IκB-α (Figure 8A–8C). However, BV-2 cells co-treated with LPS and melatonin exhibited a significant decreased in nucleus staining of NF-κB and reduced phosphorylated IκB-α protein expression (Figure 8A–8C). No observable changes in NF-κB nucleus translocation and phosphorylation of IκB-α were found in between BV-2 cells treated with melatonin alone and the control group (Figure 8A–8C).

## DISCUSSION

Growing evidence demonstrates that the BBB plays a pivotal role in the development or progression of CNS diseases [32, 33]. For instance, disruption of the BBB is associated with neuronal dysfunction, which likely contributes to onset of neurodegenerative diseases, neurotrauma and related neuropathological events such as multiple sclerosis (MS), stroke, head injury, ischaemia and reperfusion, haemorrhage, and infarction [34, 35]. The mechanisms by which the BBB breakdown occurs and progresses and the consequences are complex. Reactive oxygen species (ROS) is a common trigger that mediates further BBB compromise via a diverse of pathological pathways, including oxidative damage, TJ modification, and matrix metalloproteinases (MMPs) activation [36, 37]. Among many MMPs, MMP-9 has been studied extensively in the BBB tight junction dysfunction [38]. Emerging evidence has showed that uncontrolled inflammatory response plays a critical role in the BBB disruption resulting in neonatal WMI. Interestingly, inhibition of the CXCL5 signaling pathway [4] and TNF receptor-Jun N-terminal kinases signaling [39] significantly attenuated microglial activation, increased myelination, and reduced astrogliosis in the white matter after LPS-sensitized HII model. In the present study, we clearly demonstrate that treatment of melatonin efficiently reduces the brain damage in a rat model of HII, and this neuroprotective effect is mediated by the regulation of the BBB integrity and possibly through inhibition of the TLR4-NF-κB signaling.

The pathogenesis of WMI appears to be multifactorial. Two major risk factors are hypoxia-ischemia and its related inflammation in preterm infants [28]. These two upstream factors synergize with each other and thus amplify the detrimental effects, leading to activation of downstream mechanisms. On one hand, the brain injury caused by the HII decreases cerebral blood flow, resulting in energy depletion and the BBB disruption in white matter. On another hand, HII increases production of pro-inflammatory cytokines, gliosis, reactive oxygen species, and/or excitatory amino acids, leading to pre-OLs and axonal damage in white matter [40]. Therefore, inhibition of pro-inflammatory response as a therapeutic strategy to achieve a functional cure of WMI is proposed. In this study, our results demonstrated that increased BBB permeability and enhanced inflammatory responses are associated with LPS-induced HII leading to WMI in rats. Strikingly, treatment of melatonin significantly improved astrogliosis, microgliosis and inflammation and preserved the BBB integrity after HII in animals. Based on the *in vitro* study using a co-culture system of BV-2 cells and HUVECs, LPS-induced loss of TJ and AJ was markedly inhibited by treatment of melatonin. The anti-inflammatory effect of melatonin is likely mediated by inhibition of the TLR4/NF-κB signaling pathway. To the best of our knowledge, this is the study demonstrate that treatment of melatonin attenuates the BBB damage via inhibition of the TLR4/NF-κB inflammatory.

The BBB plays an important role in maintenance of the CNS homeostasis. The barrier function of the BBB is mainly attributed to the brain capillary ECs, which are tightly regulated by surrounding pericytes, astrocytes and neurons [41]. Adjacent ECs express continuous rows of transmembrane proteins that make homophilic contact in the intercellular space and form the TJ protein complex. The TJ protein complex seals the paracellular pathway of the BBB for various substances from the blood into the brain tissue [42]. Emerging evidence shows that neonatal WMI is accompanied by the loss of both TJ and AJ proteins [7, 33]. Our current findings are consistent with previous results showing that the disruption of the BBB is associated with the loss of TJ and AJ proteins after HII. It is known that pericytes are located next to the ECs and release a large number of endothelial permeability regulating signaling molecules. Thus the pathological role of pericytes in the regulation of the BBB integrity is currently of great interest [43, 44]. Pericytes are necessary for the formation of the BBB during embryogenesis [45] nd mice with severe loss of pericytes exhibited ECs hyperplasia, increased vessel diameter and morphological signs of increased vascular permeability [46]. Reduced protein expression of PDGFRβ and Desmin were observed in the neonatal brains after HII, whereas treatment of melatonin significantly increased PDGFRβ and Desmin protein expression in HII-induced neonatal rats. These results indicate that melatonin prevents disruption of the BBB, which is partially due to improved pericyte survival after neonatal HII.

Inflammation and glial activation are involved in the pathogenesis of neonatal and adult demyelinating disease, brain trauma, and mental disorders with observed white matter defects [47]. Glial cells, such as astrocytes and microglia, are mediators of neuroinflammation. Microglia, when is activated by exogenous or endogenous ligands, produce a number of proinflammatory cytokines, which is toxic to the CNS [48]. On one hand, astrocytes have a multitude of functions including sequestering various neurotransmitters [50], and supplying neurons with various nutrients including lactate and growth factors [49]. Furthermore, astrocytes have direct effects on ECs, and are capable of increasing the BBB permeability in response to activated and circulating T cells [51]. In the present study, we used Iba-1 and GFAP as specific markers to detect the number of microglia and astrocyte in neonatal rats after HII. Our data clearly showed that microglia and astrocytes were rapidly activated in the HII group, which were attenuated by the treatment of melatonin.

**Figure 9:**
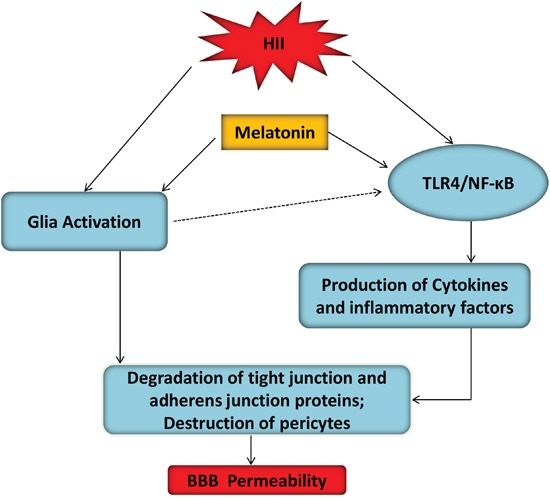
A model illustrated the BBB protective effects of melatonin after HII Glial activation and TLR4/NF-κB inflammation signaling pathway were two mutually potentiating mechanisms leading to BBB disruption in hypoxic-ischemic and inflammatory (HII) injury of the developing brain. TLR4/NF-κB signaling pathway-induced inflammation activation contributed to ECs and pericytes damage after HII, which was inhibited by melatonin.

The TLR4-mediated NF-κB signaling pathway plays a vital role in the initiation of cerebral inflammation in CNS diseases [52]. Furthermore, the activation of NF-κB leads to transcription of many pro-inflammation genes that encode cytokines, chemokines, and enzymes such as IL-1β, TNF-α and iNOS, mediators that are involved in the development of secondary brain injury following neonatal HII [13]. Activation of TLR4 signaling was suggested to be involved in LPS-induced disruption of the BBB and pharmacological inhibition of TLR4 by CLI-095 attenuated loss of occludin expression in mice with co-treatment of PCB118 and LPS [53]. Besides, VEGI attenuates the inflammatory injury and disruption of the BBB through suppressing the TLR4/NF-κB signaling pathway in animals with traumatic brain injury [54]. These studies unambiguously show that the TLR4/NF-κB signaling pathway activation is essential for mediating the BBB permeability. Previous studies showed that treatment of melatonin could exert an anti-inflammatory effect via inhibiting the TLR4/NF-κB signaling pathway in several disease models [55, 56]. Besides, melatonin promotes the BBB integrity in methamphetamine-induced inflammation in primary rat brain microvascular ECs [57]. However, the underlying signaling pathway in neonatal HII is still not fully studied. In our study, we found that administration of melatonin not only inhibited the TLR4-NF-κB signaling pathway, but also suppressed the production of inflammatory factors, such as iNOS, COX-2, TNF-α, IL-1β, IL-6 and IL-18. Furthermore, we also found that treatment of melatonin up-regulated TJ and AJ protein expressions, which are important in the maintenance of the BBB integrity in anamials after HII (Figure 9). These results indicate that treatment of melatonin could attenuate the BBB damage via modulating neuroinflammation mediated by the TLR4/NF-κB signaling pathway.

This study partly clarified the mechanisms of melatonin's neuroprotective effects on neonatal HII. But there were several limitations to our study. Firstly, in this experiment, melatonin treatment was conducted only single dose before injury, and we didn't know whether incremental dose or combined with post-injury use of melatonin would be more effective. Thus, different drug doses should be tried. Secondly, melatonin also possessed other properties including scavenging of reactive oxygen and nitrogen species, suppression of nitric oxide synthase activity, anti-autophagy, anti-apoptotic effects, amelioration of mitochondrial dysfunction, induction of antioxidative enzymes and stimulation of neuroprotective signaling pathways. Whether or not these effects also played roles in attenuation of HII, this should be further studied. Furthermore, in our following study, to further confirmed the precise mechanisms between TLR4/NF-κB signaling pathway and melatonin in BBB disruption and junction proteins regulation in HII model, TLR4 knockout rats should be applied, which contributed to the evidences of the cross-talk between TLR4 pathway and melatonin in BBB disruption and tight junction proteins regulation in HII.

In conclusion, our research demonstrated that treatment with exogenous melatonin significantly attenuated BBB permeability and degradation of tight junction and adherens junction proteins such as Occludin, Claudin-5, P120 and β-Catenin after HII, which was related to the inhibition of TLR4/NF-κB signalling pathway. Our study demonstrated that the TLR4/NF-κB signalling pathway was an important signalling cascade involved in BBB integrity and melatonin might be a new candidate as a therapeutic agent for protecting the CNS neurological diseases characterized by a compromised BBB. Thus, the present study lays the ground work for future translation of melatonin in treating neonatal brain injury in humans.

## MATERIALS AND METHODS

### HII model and melatonin administration

In this study, 10–12 postnatal day 2 (P2) Sprague–Dawley (SD) rat pups per dam were housed with a 12 h light–dark cycle and cared for according to the Guide for the Care and Use of Laboratory Animals from the National Institutes of Health and were approved by the Animal Care and Use Committee of Wenzhou Medical University. HI and inflammation were two major risk factors involved in neonatal WMI. Besides, the peak period of WMI occurrence (24-32 weeks of gestation) coincided with the highest presence of oligodendrocyte precursor in the white matter of the brain [58]. Thus, the experimental paradigm adapted from Vannucci's HI method [59] was modified to include LPS sensitization in P2 rats when the rat's brain development corresponded to 24-32 weeks of human gestational age [4]. The P2 SD rat pups were first injected intraperitoneally with 0.05 mg/kg LPS (Sigma-Aldrich, St. Louis, MO, USA) or normal saline 3 h before HI. To avoid LPS-induced body temperature change, the pups were then returned to their dams and housed in an incubator to maintain the body temperature at 33–34°C before HI. Subsequently, the left common carotid artery was permanently ligated under diethyl ether anesthesia. After 1 h recovery period, the pups were placed in an airtight glass chamber partially submerged in 36°C water baths containing a humidified atmosphere with 6.5% oxygen at a flow rate of 3 L/min for 90 min. For control measurements, a sham group was included that had a ligature placed in the identical fashion but without actually occluded the vessel, hypoxia or LPS treatment. Drug treatments were administered according to previous reports [60]. Melatonin purchased from Sigma-Aldrich (St. Louis, MO, USA) was dissolved in dimethyl sulfoxide (DMSO; Sigma-Aldrich, St. Louis, MO, USA) and diluted in saline solution to a final concentration of 5% DMSO (vehicle). All handling of melatonin was performed in the dark. Melatonin was injected intraperitoneally at a dose of 15 mg/kg at 1 h before LPS injection [60, 61]. Then, the melatonin was administered once a day for 1 week until the animals euthanized. All animals showed no significant side effects resulting from drug treatment such as an increase in mortality or infectious diseases during these experiments and the death rate of our model was zero.

### Cell culture

HUVECs were purchased from ScienCell Research Laboratories (Carlsbad, CA, USA). Cells grown in RPMI medium 1640 (Gibco, Invitrogen, Grand Island, NY) were supplemented with 10% fetal bovine serum (FBS; Gibco, Invitrogen, Grand Island, NY) and 1% penicillin/streptomycin solution (P/S). BV-2 cells were purchased from the Cell Storage Centre of Wuhan University (Wuhan, China), and were cultured in EMEM (Wisent, Quebec, Canada) containing with 10% FBS and 1% P/S. They were then incubated in a humidified atmosphere containing 5% CO_2_ at 37°C.

### Transwell co-cultures

Transwell co-cultures were performed as previously described [62]. BV-2 cells were plated onto the top side of the transwell inserts (0.4 μm pore size polyester membrane precoated with poly-L-lysine; Corning, NY, USA) at the cell density of 3 × 10^5^ cells/ml. The transwell cultures were positioned approximately 2 mm above the HUVECs-enriched cultures and the BV-2 cells grown on the transwells were separated from the HUVECs-enriched cultures by the permeable transwell membrane. Melatonin was diluted to a stock solution of 0.1 mM in 100% DMSO. Then, 1 μg/ml LPS, 100 μM melatonin, LPS plus melatonin or DMSO (100μM) was added to the media below the transwells.

### Hematoxylin–eosin (HE) staining and immunohistochemistry

At specific time points after HII, animals were anesthetized and perfused via cardiac puncture initially with 0.9% saline solution. For immunohistochemistry, animals were perfused with 4% paraformaldehyde in 0.01 M phosphate-buffered saline (PBS) after saline solution. The brains were postfixed, dehydrated using graded alcohols, embedded in paraffin, and coronal sectioned into 5 um slices. For the histological assessment of damage to the grey matter, the paraffin-embedded brain sections were stained with HE and examined under a light microscope. MBP staining for assessing myelination in the white matter was used as a marker of WMI. After the tissue hydration dewaxing and antigen retrieval at high temperature and pressure, sections were incubated with 3% H_2_O_2_/methanol for 15 min and 5% bovine serum albumin (BSA) for 30 min at room temperature [63]. Soon afterwards, we added the primary antibody against MBP (sc-13914, 1:300; Santa Cruz, CA, USA) at 4°C overnight. After triple washing in PBS, the sections were incubated with horseradish peroxidase (HRP) conjugated donkey anti-goat (sc-2020, 1:300; Santa Cruz, CA, USA) secondary antibody for 1 h at 37°C. The reaction was stopped with 3, 3-diaminobenzidine (DAB). The images were visualized using a Nikon ECLIPSE Ti microscope (Nikon, Tokyo, Japan). The results were analyzed by measurements of the integrated optical density (IOD) at ×100 or ×200 magnification using Image-pro Plus software. Immunohistochemistry was performed simultaneously in all brain samples as well as negative controls without primary antibodies.

### Evaluation of BBB permeability

BBB permeability measured by IgG extravasation staining was performed 24 h post-injury. Brain sections were incubated with 3% H_2_O_2_/methanol for 15 minutes, and then anti-IgG antibody (HRP conjugated goat anti-rat IgG, 1:100; Jackson, USA) at 4°C overnight. The reaction was stopped with DAB. Measurements of the IOD of IgG signals in the white matter were analyzed using Image-pro Plus software. The mean IOD was counted and averaged from five randomly chosen fields within each slide at ×200 magnification using a Nikon ECLIPSE Ti microscope.

### Immunofluorescence staining

Coronary brain sections were deparaffinized, rehydrated and washed twice for 7 min in PBS. For protein analysis *in vitro*, BV-2 cells were fixed for 15 min using 4% paraformaldehyde and 3 rinses in PBS. Then sections and cells were incubated with 5% BSA in PBS containing 0.5% Triton X-100 in a 37°C oven for 30 min. Then sections were incubated with antibodies against Claudin-5 (sc-28670, 1:100, Santa Cruz), CD31 (PECAM-1; sc-1506, 1:100, Santa Cruz), PDGFRβ (ab32570, 1:500, Abcam), Desmin (sc-34200, 1:100, Santa Cruz), GFAP (sc-6170, 1:100, Santa Cruz), TLR4 (ab22048, 1:500, Abcam), Iba-1 (ab5076, 1:500, Abcam) and NF-kB (p65;sc-8008, 1:100, Santa Cruz) antibody at 4°C overnight. Sections were rinsed three times in PBS after primary antibody incubation and then incubated with either fluorescent secondary antibody (1:1000, Abcam) for 1 h 37°C. The nuclei were stained with 4′, 6-diamidino-2-phenylindole (DAPI) for 5 min and finally washed in PBS and sealed with coverslips [64, 65]. All images were captured on a Nikon ECLIPSE Ti microscope (Nikon, Tokyo, Japan) at ×600 magnification.

### RT-PCR

Total RNA was isolated from fresh left hemisphere samples using the TriPure Isolation Reagent (Roche, South San Francisco, CA, USA) according to the manufacturer's instructions, and the concentration of RNA was measured by Nanodrop spectrometry (Thermo Fisher Scientific). RNA quality was determined with the OD 260/280 ratio, which was between 1.8 and 2.0. Up to 0.5μg of RNA was used to synthesize the first strand of cDNA using PrimeScript™ RT Reagent Kit (TaKaRa, Kusatsu, Shiga, Japan). Reverse transcription products were amplified with the 7900HT Fast Real-Time PCR System in a 10 μl final reaction volume using SYBR Green PCR Master Mix (Bio-Rad, Hercules, CA, USA) under the following conditions: 2 min at 95°C and 30 s at 60°C, followed by a total of 40 cycles of 2 temperature cycles (15 s at 95°C and 30 s at 60°C) [66]. The forward and reverse primer sequences are shown in Table 1. The fluorescence threshold value (Ct value) was calculated using the SDS Enterprise Database software. The Ct values of the interested genes were first normalized with β-actin of the same sample, and then the gene expression level in sham and melatonin-treated groups were calculated and expressed as fold change versus sham group (setting sham as 1).

**Table 1:** Primers used in the studies

Gene	Forward primers	Reverse primers
TNF-α	TACTCCCAGGTTCTCTTCAAGG	GGAGGCTGACTTTCTCCTGGTA
COX-2	CGGAGGAGAAGTGGGGTTTAGGAT	TGGGAGGCACTTGCGTTGATGG
iNOS	AGGCCACCTCGGATATCTCT	GCTTGTCTCTGGGTCCTCTG
IL-1β	CACCTCTCAAGCAGAGCACAG	GGGTTCCATGGTGAAGTCAAC
IL-6	GAGTTGTGCAATGGCAATTC	ACTCCAGAAGACCAGAGCAG
IL-18	AAACCCGCCTGTGTTCGA	TCAGTCTGGTCTGGGATTCGT
β-actin	AAGTCCCTCACCCTCCCAAAAG	AAGCAATGCTGTCACCTTCCC

### Western blot analysis

For western blot analysis, left hemisphere tissues were dissected and flash-frozen at −80°C. To prepare the lysates, frozen samples were weighed and minced on ice. The samples were homogenized in a modified RIPA buffer (50 mM Tris–HCl, 1% NP-40, 20 mM DTT, 150 mM NaCl, pH = 7.4) containing protease inhibitor cocktail (10 ul/ml; GE Healthcare Biosciences, PA, Little Chalfont, UK) and centrifuged at 12,000 rpm and 4°C for 10 min to collect the supernatant. Cell cultures for immunoblotting were lysed in RIPA buffer [25 mM Tris–HCl (pH 7.6), 150 mM NaCl, 1% Nonidet P-40, 1% sodium deoxycholate and 0.1% SDS] with protease and phosphatase inhibitors. The extracts above were quantified with BCA reagents. We separated proteins on a 12% gel and transferred them onto a PVDF membrane (Bio-Rad, Hercules, CA, USA). The membranes were blocked with 5% fat-free milk in TBST for 1.5 h at room temperature, and incubated with the antibodies P120 (ab92514, 1:1000, Abcam), β-Catenin (8480S, 1:1000, Cell Signaling Technology), Occludin (13409, 1:1000, Proteintech), Claudin-5 (sc-28670, 1:300, Santa Cruz), PDGFRβ (ab32570, 1:1000, Abcam), Desmin (sc-34200, 1:300, Santa Cruz), GFAP (sc-6170, 1:300, Santa Cruz), NF-κB (p65; sc-8008, 1:300, Santa Cruz), TLR4 (ab30667, 1:1000, Abcam), p-IκB-α (2859P, 1:1000, Cell Signaling Technology), IκB-α (sc-371, 1:300, Santa Cruz) and TNF-α (sc-1349, 1:300, Santa Cruz) in TBST at 4°C overnight. At last, the membrane was incubated with HRP conjugated secondary antibodies (1:10000, Bioworld) for 1 h at room temperature [67, 68]. Signals were visualized by ChemiDocXRS + Imaging System (Bio-Rad). β-actin (AP0060, 1:3000, Bioworld) was used as an internal control. Experiments were repeated three times and the densitometric values of the bands on western blots obtained by Image J software (National Institutes of Health, Bethesda, MD, USA) were subjected to statistical analysis.

### Statistical analysis

All data were expressed as mean ± SEM. The statistical analyses were performed by one-way analysis of variance (ANOVA) followed by Dunnett's multiple comparison test. A probability level of *P* < 0.05 was set as statistically significant. Each experiment consisted of at least three replicates per condition.
